# Effect of Alkaline Salt Stress on Photosynthetic Activities of Potato Plants (*Solanum tuberosum* L.)

**DOI:** 10.3390/plants14192979

**Published:** 2025-09-26

**Authors:** Congang Shen, Wenhui Yang, Yichen Kang, Shuhao Qin, Weina Zhang, Yuhui Liu, Siyuan Qian, Yuchen Han

**Affiliations:** State Key Laboratory of Aridland Crop Science, College of Horticulture, Gansu Agricultural University, Lanzhou 730070, China; 13733170735@163.com (C.S.); y2478626991@126.com (W.Y.); qinsh@gsau.edu.cn (S.Q.); zhangwn@gsau.edu.cn (W.Z.); lyhui@gsau.edu.cn (Y.L.); qiansy202503@163.com (S.Q.); 13939222103@163.com (Y.H.)

**Keywords:** potato (*Solanum tuberosum* L.), alkaline salt stress, fluorescence parameters, chlorophyll, photosynthesis

## Abstract

Land salinization severely limits the development of agriculture, and the growing global population poses a serious challenge to food security. As an abiotic stress factor limiting photosynthesis in potatoes (*Solanum tuberosum* L.), alkaline salt stress significantly impacts their photosynthetic activity. In this study, potted seedlings of the ‘Atlantic’ variety were planted in the pots. Sodium bicarbonate (NaHCO_3_) was incorporated into the dry soil within the pots at four distinct concentration levels: 0 mmol/L, 20 mmol/L, 40 mmol/L, and 60 mmol/L. The findings indicated that at a concentration of 60 mmol/L, the initial fluorescence (*Fo*) exhibited its peak value. At this concentration, NaHCO_3_ stress induced a significant decline in several parameters: variable fluorescence (*Fv*), the chlorophyll fluorescence ratio (*Fv*/*Fm*), dark-adapted maximum fluorescence (*Fm*), the *Fv*/*Fo* ratio, and overall plant performance. Compared to the control CK, the values of *Fv*, *Fv*/*Fm*, *Fm*, and *Fv*/*Fo* decreased by 42.36%, 20.44%, 54.1%, and 61.97%, respectively. At a stress concentration of 60 mmol/L, NaHCO_3_ stress exhibited a more pronounced inhibition of chlorophyll synthesis. Under T3 treatment at this stress concentration, the contents of chlorophyll **a**, chlorophyll **b**, and total chlorophyll **a**/**b** were significantly lower than the control group (CK), decreasing by 46.29%, 54.3%, and 48.56%, respectively. The T2 treatment showed the next most pronounced reduction, with levels 33.26%, 45.75%, and 36.79% lower than CK, respectively. After a brief increase in the intercellular CO_2_ concentration (Ci) in photosynthetic gas exchange, the net photosynthetic rate (Pn), stomatal conductance (Gs), and transpiration rate (Tr) decreased significantly with the gradual increase in concentration and prolongation of time. The expression levels of genes related to some subunits of photosystem II and photosystem I were down-regulated under stress, while the expressions of genes related to Fd and FNR were also down-regulated to varying degrees. In this study, photosynthetic activities such as fluorescence parameters, chlorophyll content, and photosynthetic gas exchange were measured, along with 16 key photosynthetic genes of potato plants. The aim was to explore the effects of alkaline salt stress on potato photosynthesis and its related mechanisms. The research outcomes contribute to a better understanding of potato’s adaptive responses to alkaline stress, potentially informing future efforts in crop improvement and saline agriculture management.

## 1. Introduction

Soil salinisation is one of the abiotic stress factors limiting plant growth and development. It severely impacts the sustainable development of agricultural ecosystems. Globally, approximately 20.715 billion acres of land are affected by salinisation. China’s total saline-alkali land area reaches 105 million hectares, with 6.3% of its arable land classified as saline-alkali farmland [1]. Based on varying salinity concentrations, saline-alkali soils are categorised into mild, moderate, and severe types. Northwest China represents a concentrated distribution zone for moderately to severely saline-alkali soils [2,3]. This region enjoys abundant sunlight and extended photosynthetic periods for plants, yet its arid conditions and sparse rainfall characterise it as a semi-arid rain-fed agricultural zone. This environment poses significant challenges to crop cultivation.

Potato (*Solanum tuberosum* L.) has emerged as a priority crop in the arid and semi-arid regions of China, owing to its resilience to abiotic stresses and its dual utility as a staple food and a vegetable [4]. As the world’s third-largest food crop after rice and wheat, global annual potato production averages 374 million tonnes. China ranks as a major global potato producer, accounting for one-quarter of worldwide output. This achievement stems from government initiatives promoting potato as a staple food, alongside maintaining the world’s largest potato cultivation area since 2007 [5]. However, with China’s population pressure increasing annually, food supply issues have become increasingly prominent. Concurrently, the persistent deterioration of the environment remains a key factor constraining the development of the potato industry. Furthermore, most related research has focused on plant salt stress, while studies on alkaline salt stress in potatoes remain relatively scarce [6].

Sodium bicarbonate (NaHCO_3_), also known as baking soda, has a pH concentration higher than NaCl and is an alkaline salt. Compared with neutral salts, alkaline salts are more invasive to plants, and soil salinization is also one of the key environmental factors hindering the development of the potato industry [7]. Alkaline salt stress is mainly manifested in osmotic stress, ionic toxicity, and difficulty in water and nutrient absorption. Studies have shown that plant roots in saline-alkali soils are highly susceptible to higher ion poisoning, and studies taking NaHCO_3_ as an example have shown that HCO_3_^−^ ions are the main mechanism leading to ion poisoning of plants, and at the same time disrupt the mutual exchange of nutrients and water absorption among roots, stems, and leaves [8,9,10]. However, long-term alkaline salt stress on potato roots inhibits shoot and leaf growth. The resulting nutrient deficiency in cells leads to light inhibition, which hinders photosynthesis and disrupts the thylakoid membranes in leaf cells. Additionally, chlorophyll content decreases, while high pH and ion concentrations damage both PSI and PSII, disrupting the regulatory balance between them. This stress also induces photolysis and abnormal photosynthetic electron transport, impairing the synthesis of adenosine triphosphate (ATP) and reduced nicotinamide adenine dinucleotide phosphate (NADPH) [8,11]. Consequently, these effects increase the risk of photosynthetic system dysfunction and lead to stunted growth and development in crops. Chlorophyll fluorescence parameters are very important in plant physiology research, which can reveal the dynamic changes in photosynthetic system in real time, especially in the study of plant growth regulation and stress resistance. The chlorophyll fluorescence parameter can accurately express the stress damage of the PSII reaction centre, and the chlorophyll fluorescence parameter of plants will change significantly when subjected to salinity–alkali stress, which also shows its importance in the study by Alkahtani et al. [12]. On the one hand, in order to protect the photosynthetic system of plants from being harmed by excessive light energy, the stress resistance strategy of plants by dynamically adjusting fluorescence parameters will be triggered under abiotic stress, and plants will activate corresponding defence mechanisms in which non-photochemical quenching (NPQ) may increase and excess light energy will be lost in the form of heat energy [11]. In other aspects, small molecules accumulate in plant cells, among which the increase in proline and betaine content can effectively maintain cell turgor pressure and ensure the normal metabolism, respiration, and photosynthesis of cells in crops [13,14,15,16]. Thylakoids are an important site for photosynthesis, and the chlorophyll content determines the intensity of photosynthesis in plant leaves, which affects the yield of crops to a certain extent [17]. Under alkaline salt stress conditions, potato leaves will also initiate a series of salt–alkali resistance characteristics [8]. Plants will adjust the proportion of chlorophyll content to cope with the loss of photosynthetic energy in the stress environment, and the spectral range of chlorophyll **b** absorption is greater than that of chlorophyll **a**, especially under low light and astigmatism [18]. Chlorophyll **a** plays a key role in the collection and transfer of light energy in photosynthetic reactions and is also a participant in the construction of the photosystem, which works with respiration to maintain photosynthesis in plants [19]. On the other hand, salt stress inhibits the expression of genes associated with photosynthesis in plants. Zhao et al.’s research has revealed that under saline–alkali treatment conditions, the expressions of the PSI gene psaA and Calvin cycle genes in seashore mallow (*Kosteletzkya pentacarpos* L.) are suppressed. Differentially expressed genes (DEGs) predominantly exhibit down-regulation, leading to an imbalance in the ATP/NADPH ratio [20]. Research into genes related to photosynthesis in potatoes is essential. Similar reports indicate that, compared to salt stress, alkaline salt stress significantly inhibits the expression of the chlorophyll synthesis gene chl H and the photosystem II photosynthetic reaction gene psbO in grapevines (*Vitis vinifera* L.). This leads to a 40% reduction in chlorophyll content and a decrease of 0.2 units in photochemical efficiency (*Fv*/*Fm*) [21]. Therefore, investigating the effects of alkaline salt stress on the photosynthetic activity of potato plants and the genes associated with photosynthesis is of paramount importance for elucidating the physiological mechanisms underlying potato responses to alkaline salt stress. This study can not only reveal the stress response mechanism and provide a theoretical basis but also provide important scientific guidance for the follow-up experiments of high-yielding and stable-yielding potato and help the research and development of efficient potato cultivation technology and variety improvement in saline–alkali environments.

## 2. Results

### 2.1. Effects of Different Concentrations of NaHCO_3_ on Potato Fluorescence Parameters

**Figure 1:**
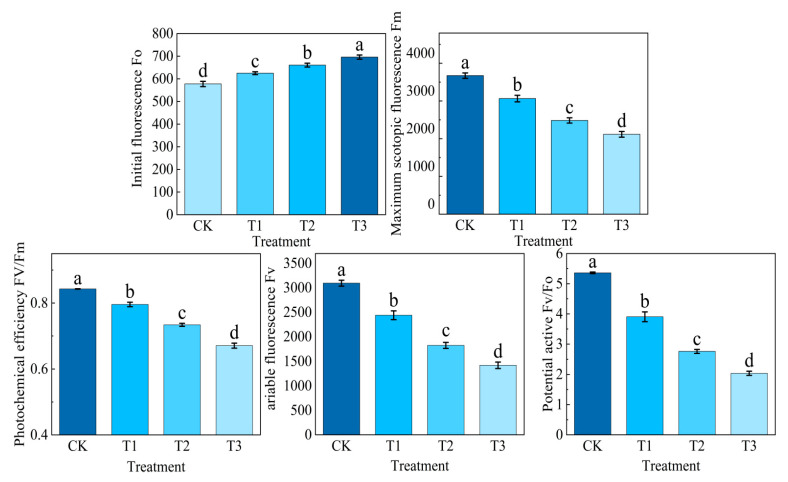
Effects of different concentrations of sodium bicarbonate treatment on chlorophyll fluorescence parameters in potato leaves. **Note:** Error bars refer to ± standard error. Different small letters above the error bar indicate a significant difference between each treatment at the *p* < 0.05 level.

It can be seen from Figure 1 that the fluorescence parameters of potato potted seedlings showed different degrees of change after stress, and the initial fluorescence *Fo* value increased with the increase in NaHCO_3_ concentration, and the initial fluorescence *Fo* increased to different degrees under each treatment compared with CK, and the initial fluorescence *Fo* under T3 treatment was significantly higher than that of CK by 20.58%. T2 treatment was second only to T3 treatment, which was 14.43% higher than that of CK. The dark-adapted maximum fluorescence parameters *Fm*, photochemical efficiency parameters *Fv*/*Fm*, variable fluorescence parameters *Fv*, and the *Fv*/*Fo* ratio of each treatment decreased to varying degrees compared with CK, and the dark-adapted maximum fluorescence parameters *Fm*, photochemical efficiency parameters *Fv*/*Fm*, variable fluorescence parameters *Fv*, and the *Fv*/*Fo* ratio were the smallest under T3 treatment, which were significantly lower than those of CK by 42.36%, 20.44%, 54.1%, and 61.97%, respectively. The T2 treatment was second only to the T3 treatment, which was 32.34%, 12.93%, 41.11%, and 48.52% lower than that of CK, respectively.

### 2.2. Effects of Different Concentrations of NaHCO_3_ on Potato Chlorophyll

**Figure 2:**
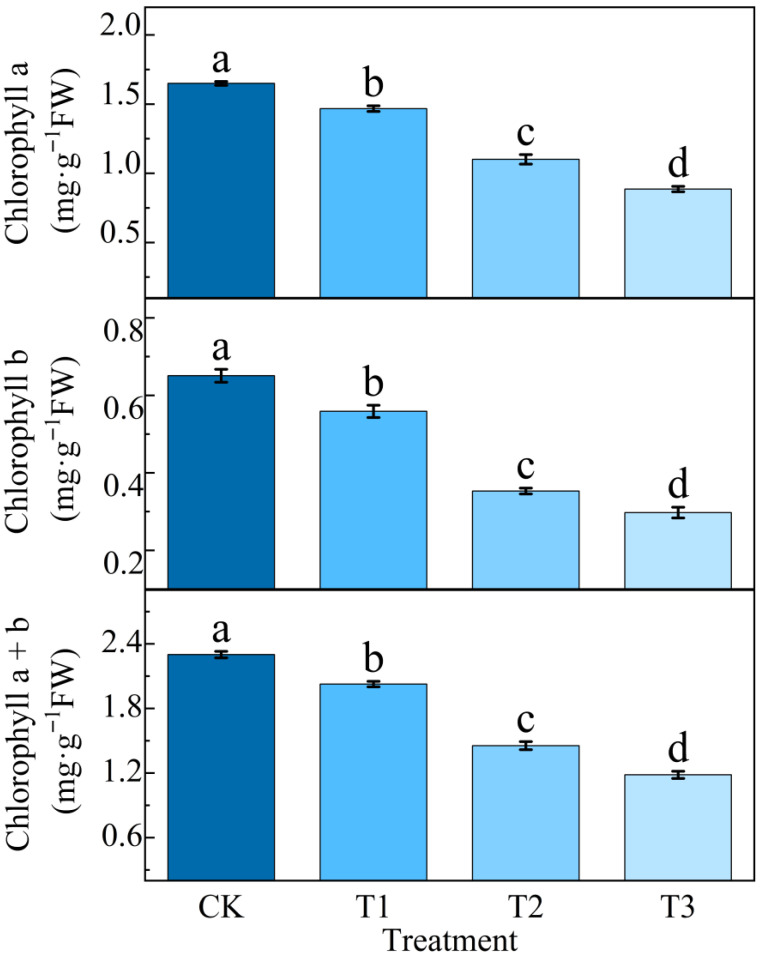
The impact of alkaline salt stress under different concentration gradients on potato chlorophyll.

As shown in Figure 2, there was a significant difference between the change in chlorophyll content and CK, and when the stress concentration reached 60 mmol/L, the inhibition of chlorophyll synthesis by NaHCO_3_ stress was more serious, and the chlorophyll content of the potato plant decreased to varying degrees with the increase in stress concentration. Chlorophyll **a**, chlorophyll **b,** and chlorophyll **a**/**b** under T3 treatment were significantly lower than those in CK, which decreased by 46.29%, 54.3%, and 48.56%, respectively. The T2 treatment was second only to T3, which was 33.26%, 45.75%, and 36.79% lower than that of the CK treatment, respectively.

### 2.3. Effects of Different Concentrations of NaHCO_3_ on Potato Photosynthesis

**Figure 3:**
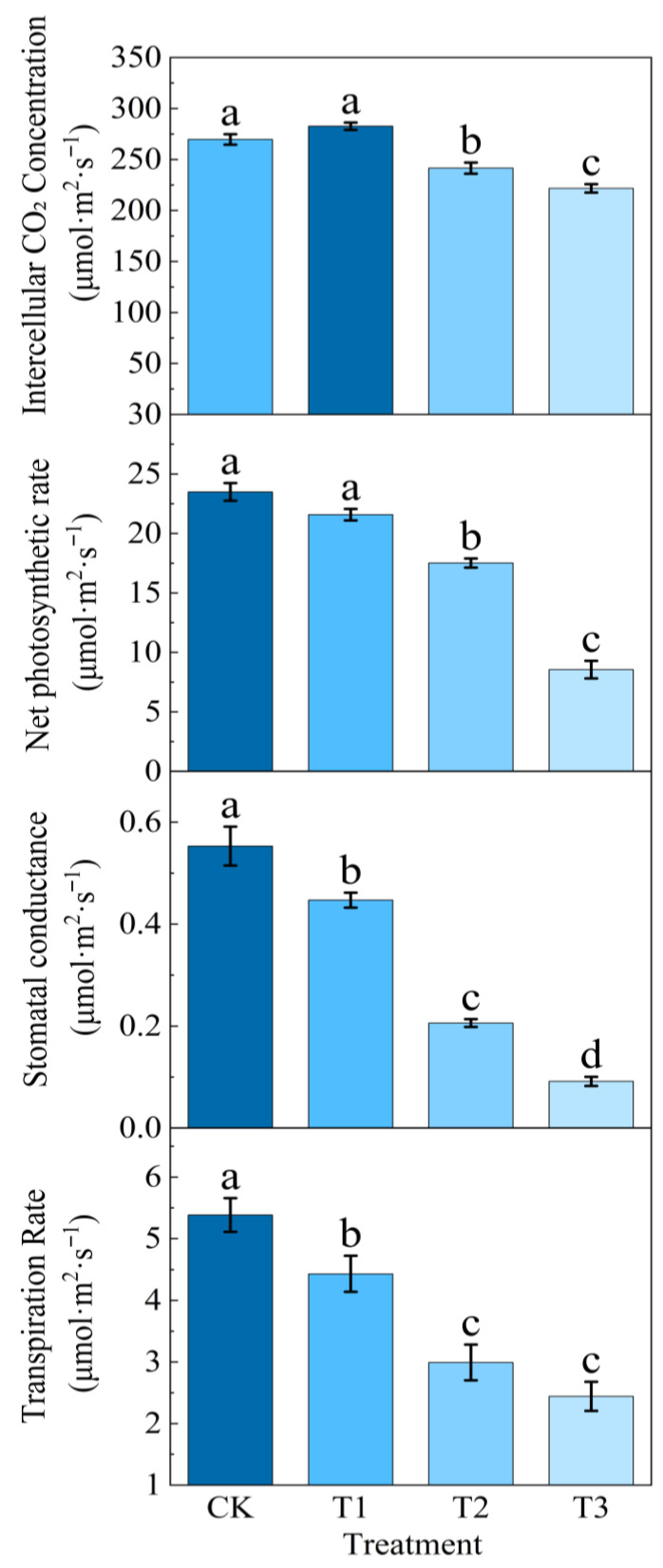
Effects of alkaline salt stress on photosynthetic parameters of potato under different concentration gradients.

According to Figure 3, the intercellular CO_2_ concentrations under photosynthetic treatments increased first and then decreased, with T1 treatment increasing by 4.8% compared with CK, and T2 and T3 treatments decreasing to varying degrees compared with CK, which were 10.45% and 17.78%, respectively. The net photosynthetic rate, stomatal conductance, and transpiration rate under the stress treatment showed a downward trend with the increase in stress concentration, and the net photosynthetic rate, stomatal conductance, and transpiration rate under T3 treatment were the smallest, which were significantly decreased by 63.6%, 83.44%, and 54.64%, respectively, compared with the CK treatment. T2 was second only to T3, which was 25.45%, 62.76%, and 44.41% lower than CK, respectively.

### 2.4. Effect of NaHCO_3_ on the Expression of Key Genes for Potato Photosynthesis

In the alkaline salt stress environment, not only is a high salt concentration usually accompanied by a high pH value, but it also belongs to compound stress. We observed that the 16 selected genes exhibited varying degrees of down-regulation following exposure to alkaline salt stress, indicating that the impact of stress on photosynthesis-related genes in potatoes may be more severe.

**Figure 4:**
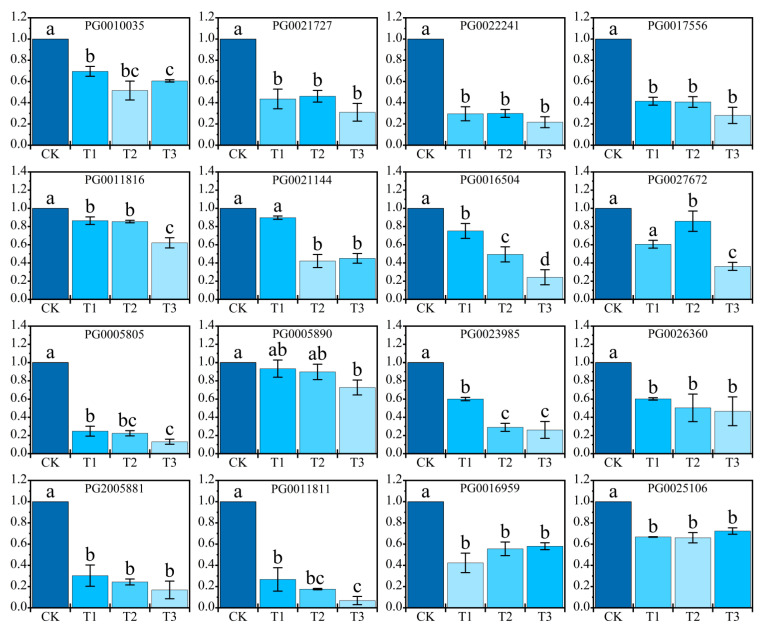
Expression of key genes of potato photosynthesis under NaHCO_3_ stress. **Note.** PG is the abbreviation of PGSC0003DMG40. The same below.

As shown in Figure 4, the photosynthesis of potato plant involves the expression of multiple genes, and the relative expression levels of psbO (PG0010035)-related genes under T3 treatment increased to a certain extent compared with T1 and T2 treatments with the increase in concentration under alkaline salt stress, but not significantly, and the relative expressions of T1 and T2 were down-regulated compared with the CK treatment. The relative expression levels of PsbQ- and PsbR-related genes (PG0021727 and PG0022241) of PSII were significantly down-regulated under NaHCO_3_ stress. The expression levels of psbS-related genes (PG0017556) in PSII did not change significantly in T1 and T2 treatments after alkaline salt stress, and T3 treatment decreased significantly compared with CK. The expression levels of psaD and psaF (PG0011816 and PG0021144) in PSI decreased with the increase in stress concentration, and the expression of each treatment decreased compared with CK. The relative expression levels of PsaL-related genes (PG0027672) of PSI subunit PsaL decreased–increased–decreased, and the T2 treatment was significantly higher than that of other treatments except CK, and CK was significantly different from that of the T3 treatment. Compared with each treatment, CK was significantly different in the expression of PsaN (PG0005805) in the subunit of PSI, and CK was higher than that of the T1, T2, and T3 treatments. However, in the related genes (PG0005890) of psaO, the expression of CK was lower than that of the T1 treatment, but not significantly, and the expression of T2 was not significantly lower than that of the T1 treatment. The relative expression levels of photosynthetic electron transport Fd (PG0023985, PG0026360, PG2005881)-related genes decreased with the increase in NaHCO_3_ concentration, and the lowest value was when the concentration reached 60 mmol/L. The subunit-related genes (PG0016959) of F-type H transporting ATPase showed a downward trend first and then an upward trend, with T3 second only to the CK treatment, and the relative expression level of the T1 treatment was the lowest. Another F-type H transports ATPase subunit-related genes (PG0025106), and when the concentration of NaHCO_3_ stress was 40 mmol/L, the T2 treatment had the lowest value, the T1 treatment value was higher than T2 but not obvious, and the T3 treatment value was second only to CK.

### 2.5. Potato Photosynthetic Pathway Diagram Under NaHCO_3_ Stress

**Figure 5:**
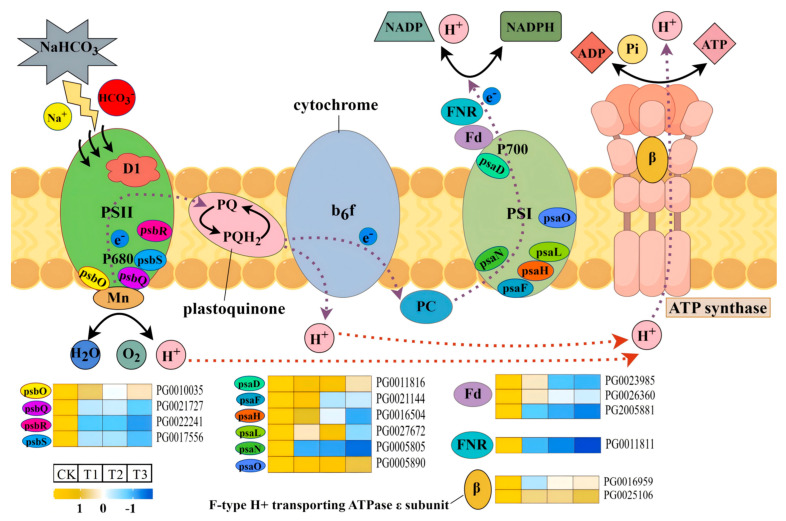
A model shows the changes in photosynthesis-related genes in potatoes after alkaline salt stress.

Compared with salt stress, alkaline salt stress significantly increased the damage to plant photosynthesis. In order to understand the mechanism of potato plants’ photosynthesis under alkaline salt stress, as indicated in Figure 5, we created a model in which the damage of subunit-coding genes in the photosystem was significantly increased by the increase in alkaline salt stress concentration, and the relative expression of FNR-related genes (PG0011811) was significantly down-regulated. When the high concentration of ions in the NaHCO_3_ solution enters the cells of potato stems and leaves, CO_2_ accumulates briefly in the early stage of stress, decreases significantly in the later stage, and the photosynthetic system collapses completely. A large amount of HCO_3_^−^ and Na^+^ ions penetrate the cell body, destroying the cell membrane structure, and the accumulation of reactive oxygen species (ROS) attacks the photosystem D proteins, resulting in a sharp decrease in chlorophyll content, which is positively correlated with the down-regulation of PSII-related genes. When the light energy is absorbed by the antenna protein, it is transferred to P680 in the reaction centre of PSII, which will be decomposed into O_2_ and H^+^, which will be passed to the cytochrome b_6_f complex through plastoquinone (PQ) and transferred to Fd in PSI to reduce NADP^+^ to NADPH, and finally synthesised to ATP through the combined subunit processing of ATPase and refluxed back to the chloroplast matrix along the concentration gradient. As an important component of photosynthesis, the OEC (oxygen release complex) of PSII is involved in the photolysis and oxygen release process of water and the decrease in PSII. The decrease in maximum photochemical quantum yield (*Fv/Fm*) was positively correlated with the down-regulation of the expression of psbQ-, psbR-, and psbS-related genes (PG0021727, PG0022241, and PG0017556) on PSII. The genes related to photosynthetic electron transport were also affected by HCO_3_^−^ and Na^+^, and the genes related to Fd (PG0023985, PG0026360, PG2005881) and FNR (PG0011811) were down-regulated to varying degrees.

## 3. Discussion

As a typical abiotic stress factor, saline–alkali stress occupies an important position in a variety of environmental stresses faced by agricultural production, which seriously affects the growth and development of crops and the composition of yield [22]. Compared with NaCl, the pH value of the NaHCO_3_ solution was higher, the ionic toxicity caused by the NaHCO_3_ solution to plant photosynthesis was more significant, and alkaline salt stress synergistically inhibited potato photosynthesis through the dual stress mechanism of osmotic stress and ionic toxicity, which was manifested in the significant down-regulation of the expression of photosynthetically related genes such as a decreased photosynthetic rate, blocked chlorophyll biosynthesis, imbalance between stomatal conductance and intercellular CO_2_ concentration, and decreased Rubisco enzyme activity. When a large amount of HCO_3_^−^ and Na^+^ in the alkaline salt solution NaHCO_3_ enter the cell, Na^+^ will destroy the lipid bilayer structure of the thylakoid membrane, and the excessive accumulation of HCO_3_^−^ in the cell exceeds the capacity of the cell, resulting in a loss of the chloroplast’s ability to capture light, significantly inhibiting the activity of Chl a and Chl b synthases, resulting in the blockage of chlorophyll synthesis [23,24,25]. In this experiment, with the increase in NaHCO_3_ concentration, the decrease in the net photosynthetic rate, stomatal conductance, and transpiration rate was positively correlated with chlorophyll, and Li et al. [26] studied that the negative effect of alkaline stress on alfalfa seedlings was stronger than that of salt stress, and the photosynthetic parameters and chlorophyll content decreased significantly. It was verified that environmental factors such as a high pH value under alkaline salt stress severely restricted plant growth and development, photosynthesis, and crop yield through related pathways [27,28].

Some scholars have analysed the photosynthetic mechanism of chloroplasts in response to NaHCO_3_ stress, which weakens the stability and electron transport efficiency of the PSII and PSI core protein complexes by destroying the structural integrity of the thylakoid membrane of Suaeda salsa, and reducing the linkage [29]. In the photosynthesis pathway diagram of this study, photosynthesis is initiated from PSII, and chlorophyll absorbs light energy and converts it into electrical energy. In this process, high concentrations of HCO_3_^−^ increase the pH environment on the outer side of the thylakoid membrane, interfere with the interaction between the oxygen-releasing enhancers psbO and psbQ subunits and OEC, and thereby inhibit the photolysis of water and the release of oxygen. When PQH_2_ (plastoquinol) transfers electrons to the cytochrome b_6_f complex, its efficiency in driving proton transport across membranes through energy conversion decreases significantly. In the subsequent PSI stage, psaF was the key subunit that bound to plasticocyanin (PC), and the expression of related genes (PG0021144) decreased due to the influence of a high concentration of HCO_3_^−^, which reduced its affinity with PC and significantly affected the electron transport efficiency. Berry et al.’s research on (*Chlamydomonas reinhardtii* L.) demonstrated that PsaF is an essential subunit for the transfer of electrons from PC to PSI, and it is an important part of photosynthesis [30,31]. Fd and FNR are the key electron transfer proteins in the photoreaction, Fd is responsible for receiving electrons from PSI to regulate the electron flow direction and reducing power, and it is combined with FNR ferredoxin-NADP reductase to co-generate NADPH to prepare for carbon fixation in the dark reaction, and the relative expression levels of Fd (PG0023985, PG0026360, PG2005881)-related genes in this experiment were the lowest values of the T3 treatment when the concentration of NaHCO_3_ was 60 mmol/L. The results indicate that the expression of related genes in Fd, as the core vector of photoreaction transmission, was seriously down-regulated after alkaline salt stress. Similarly, Subramanyam’s study demonstrated that exposure of Chlamydomonas reinhardtii to 100 mM NaCl caused (*Chlamydomonas reinhardtii* L.) cells to be significantly damaged at a concentration of 100 mM NaCl, and the results showed that the psaD, psaE, and psaF subunits of the PSI reaction centre were significantly reduced under high salt stress, the binding sites of Fd were destroyed, and the electron transport rate was significantly reduced [32]. In addition, the decrease in the electron transport rate of Fd directly affects the catalytic activity of FNR. FNR is another key photosynthetic enzyme that promotes photosynthesis, and Fd transfers electrons to FNR to catalyse the reduction of NADP^+^ to NADPH [33,34]. In this study, the expression level of the FNR-related gene (PG0011811) in the T3 treatment was significantly different from that of the control treatment, the expression level of the T3 treatment was the minimum, and the expression level was significantly down-regulated. We observed that the expression of genes associated with the subunits psaD (PG0011816), psaF (PG0021144), psaH (PG0016504), psaL (PG0027672), and psaN (PG0005805) was down-regulated in this trial. Quantitative proteomic analysis of salt stress in rice (*Oryza sativa* L.) by Xu et al. found a 1.585-fold increase in psaD-subunit-related protein in photosynthetic system I, with a 37.6% decrease in the level of the psaH subunit-related protein [35]. Li et al. studied that PsaN and PsaO, the PSI-related subunits of (*Cymbidium ensifolium* L.), were significantly down-regulated at the transcriptional level in response to salt stress in the leaves, and the down-regulation was amplified with the duration of stress [36]. Such reports demonstrate the level of damage to plant photosynthetic system subunits in saline–alkali environments. Furthermore, relevant research findings indicate that plant photosynthesis-related genes continue to exert positive effects under adverse conditions [37,38]. Among these, the light-responsive transcription factor SlBBX20 in tomatoes enhances the plant’s tolerance to saline–alkali stress. It increases the net photosynthetic rate and leaf pigment content, thereby maintaining the photosynthetic capacity of the foliage [39].

Fluorescence parameters are important indicators to characterise the light use efficiency of plants under stress and are a favourable tool to detect the stress level of plants. Previous studies have shown that the chlorophyll fluorescence of lettuce gradually decreases with increasing concentrations of NaCl under salt stress, reaching its lowest values at a concentration of 400 mM [40,41]. In this experiment, the fluorescence parameters decreased significantly with the increase in NaHCO_3_ concentration and the extension of time, *Fv* variable fluorescence, *Fv*/*Fm* photochemical efficiency, *Fm* dark adaptation maximum fluorescence, and *Fv*/*Fo* potential activity. In this experiment, when the concentration of NaHCO_3_ reached 60 mmol/L, the T3 treatment in *Fv*, *Fv*/*Fm*, *Fm*, and *Fv*/*Fo* was the minimum; the increase in the initial fluorescence value from low to high indicated that the increase in stress concentration deepened the degree of SPII damage, the increase in Fo as an early signal of PSII functional damage was closely related to the burst of ROS (reactive oxygen species) [42], and the strong oxidation of ROS would accumulate in large quantities and attack the response centre of SPII. Based on the damage of the photosynthetic system, in which the D1 protein is extremely sensitive to ROS, this process leads to an inability of the excited state energy of P680 to be transmitted to QA (plastidoquinone) through charge separation but is released in the form of fluorescence (*Fo*) to increase, in addition, under alkaline stress conditions. PsbO in PSII is the earliest damaged subunit, and the down-regulation of the relative expression of its encoded gene (PG0010035) will significantly affect the normal level of *Fo*. Studies have shown that at a pH above 7.5, PsbO dissociates from PSII due to environmental factors, resulting in an imbalance in OEC activity and an increase in *Fo* [43,44]. The degree of damage to the reaction centre of PSII was positively correlated with the change in Fv, which directly reflected the degree of damage to the photosynthetic mechanism by alkaline salt stress, and in our study, *Fv* decreased significantly when the concentration of NaHCO_3_ reached 60 mmol/L compared with the pretreatment under stress, and Zhang et al. studied the damage of the photosystem of sorghum under different types of sodium salt and alkaline salt stress, confirming the positive correlation between the change in Fv and the degree of PSII damage [45]. The essence of *Fm* is to reflect the fluorescence yield when the PSII reaction centre is completely closed and is usually used to judge the potential activity of PSII and the functional state of the photosynthetic mechanism. In this experiment, when NaHCO_3_ was 60 mmol/L, *Fm* had the lowest value, and the decrease in Fm indicated that the photosynthetic device was damaged. *Fm* reflects the light energy harvesting ability of the PSII antenna system and is positively correlated with the total chlorophyll content, chlorophyll **a** and chlorophyll **b**; the higher the total chlorophyll value, the greater the *Fm* value. In addition, previous studies have similarly reported that the chlorophyll fluorescence index *Fv*/*Fm* alkali stress of grape leaves was significantly lower than that of salt stress, and the chlorophyll content was also significantly reduced, indicating that the photosynthetic apparatus was damaged, the photoenergy conversion efficiency of the PSII reaction centre was reduced, and the photosynthetic rate was reduced [21].

The decrease in Pn will lead to a significant decrease in the photosynthetic carbon assimilation ability of potato leaves, and the insufficient energy supply will further lead to a decrease in the synthesis of ATP and NADPH, which will affect cellular energy metabolism and further affect the yield of potato, which is manifested in the reduction in starch accumulation in potato tubers [46]. Relevant studies indicate that changes in the net photosynthetic rate are primarily caused by non-stomatal limiting factors. Kang et al. showed that the short-term drought on the 40th day after sowing had the strongest inhibitory effect on Pn under salt stress, but salt stress had a long-term impact on peanut yield [47,48]. The results of Liu et al. showed that, except for the intercellular carbon dioxide concentration, the net photosynthetic rate, stomatal conductance, and transpiration rate of *Leymus chinensis* leaves decreased under saline–alkali stress. Under alkaline salt stress, *Leymus chinensis* leaves absorb a large amount of ions, the Na^+^ mass fraction in leaves increases, and in order to ensure that water loss is reduced, plants will actively close their stomata to ensure that water loss is reduced, and other triggers include increasing abscisic acid content, which acts as a signalling molecule to promote stomatal closure [49]. NaHCO_3_ stress inhibited photosynthesis and caused carbon metabolism disorders in plants, especially the inhibition of Rubisco (ribulose-1,5-bisphosphate carboxylase/oxygenase). As a key rate-limiting enzyme of the Calvin cycle, the decrease in Rubisco activity directly leads to a decrease in carbon assimilation efficiency, which in turn affects the synthesis and transport of photosynthetic products. Similarly, Zhang et al. showed that NaCl treatment delayed the growth and development of wheat seedlings, accompanied by a decrease in starch content and a decrease in Rubisco-related enzyme activity [50,51]. In this experiment, the stomatal conductance decreased with the increase in NaHCO_3_ stress concentration. Li et al. showed that under the combined stress of salt and heat, the stomatal conductance of tomato leaves decreased significantly more than that under single stress, resulting in a sharp decrease in the photosynthetic rate. Metabolomic analysis showed that the imbalance in carbon metabolism caused by stomatal closure further inhibited the expression of photosynthetic genes [52]. Stomata are the main channel of transpiration, and when the stomata are closed, the transpiration rate decreases significantly. The stomatal conductance is positively correlated with the transpiration rate, and we observe that when the concentration of NaHCO_3_ reaches 60 mmol/L, the stomatal conductance and leaf transpiration rate are the minimum values. Shen et al. found that in an environment of low salt stress, plants can alleviate stress through the accumulation of osmotic regulatory substances, and once the stress concentration reaches the critical threshold (100 mmol L^−1^), this mechanism will fail, and photosynthetic parameters such as stomatal conductance and transpiration rate will begin to decrease [53]. In this study, the growth environment of potato under alkaline salt stress was artificially simulated, and the physiological indices of photosynthesis were systematically analysed, aiming to elucidate the stress mechanism of alkaline salt stress on potato photosynthesis and to provide an innovative direction for guiding the development of new saline–alkali-tolerant varieties in the future.

## 4. Materials and Methods

### 4.1. Experimental Design

The ‘Atlantic’ variety was selected as the primary container-grown experimental material due to its known sensitivity to abiotic stress and its suitability for processing. The trial was conducted from May to September 2019 on the campus of Gansu Agricultural University (longitude: 103.70; latitude: 36.09). Due to the risk of field contamination from the application of alkaline salt stress, the experiment employed outdoor pot cultivation. The specifications are 30 cm × 35 cm, and the culture medium is mixed from vermiculite and perlite in a 4:1 volume ratio. Before sowing, disinfect the substrate with carbendazim and ensure that the substrate is the same height as the pot; water thoroughly two days before sowing, apply basal fertiliser the day before, screen out the full bud body, and sow the potato seed, placing one seed potato in each pot with the sprout facing upwards, and set up the seedlings after shading with a sunshade net and other routine management. In this experiment, four different gradients of NaHCO_3_ concentration treatment were established, namely CK: 0 mmol/L, T1: 20 mmol/L, T2: 40 mmol/L, and T3: 60 mmol/L, with each treatment set up in three replicates. All treatments were poured according to the designed NaHCO_3_ concentration, poured once every 7 days, and samples were taken for the determination of physical and chemical properties after 30 days of stress treatment.

### 4.2. Potato Determination of Fluorescence Parameters

The fluorescence parameters were determined by Li-6400XT (LI-COR Biosciences, Lincoln, NE, USA) photosynthetic instrument (equipped with fluorescence leaf chamber), and the 3rd and 4th leaves at the top of the plant were selected for potato measurement. The photosynthetic parameters were measured from 8:50 a.m. to 11:00 a.m., the LED light intensity was controlled at 860 μmolm^−2^ s^−1^, and the temperature was 25 ± 1 °C. The determination of fluorescence parameters was carried out after 22:30 in the evening, under the condition of ensuring that there was no strong light source. The specific method referred to Gao’s [54] method, and the measurement indexes were as follows: initial fluorescence, *Fo*; variable fluorescence, *Fv*; photochemical efficiency, *Fv/Fm*; dark adaptation maximum fluorescence, *Fm*.

### 4.3. Potato Determination of Chlorophyll Content

The chlorophyll content was determined by spectrophotometry. In total, 2 g of potato leaves were taken from each sample, and three biological replicates were performed. The chlorophyll content in the plants was determined using the chlorophyll content detection kit (BC0990). This kit was provided by Beijing solarbio Co., Ltd. (Beijing, China), and the test method can be referred to in the kit’s user manual.

### 4.4. Potato Determination of Photosynthetic Gas Exchange

The photosynthetic gas exchange parameters were determined by the Li-6400XT photosynthetic instrument (LI-COR Biosciences, Lincoln, NE, USA), equipped with a fluorescence leaf chamber from 9:00 a.m. to 11:00 a.m., and the measurement indices were as follows: net photosynthetic rate (Pn), stomatal conductance (Gs), intercellular CO_2_ concentration (Ci), transpiration rate (Tr). The photosynthetic parameters were measured from 9:00 a.m. to 11:00 a.m. During the measurement, the LED self-set light intensity was controlled at 800 μmol·m^−2^ s^−1^, a CO_2_ concentration of (390 ± 10) mol·L^−1^, with a relative humidity of 45–50%, and the determination method referred to Zhang’s method [55].

### 4.5. RNA Extraction, Reverse Transcription, and Quantitative Real-Time Polymerase Chain Reaction

Referring to the previous study [7], 16 differentially expressed mRNAs were selected in this study. Total RNA was extracted using the SteadyPure Universal RNA Extraction Kit (Accurate, Changsha, China), and mRNA was reverse transcribed using FastKing gDNA Dispelling RT SuperMix (Tiangen, Beijing, China). Actin was used as an internal reference gene, and qRT-PCR was verified on a QuantStudio 6 Flex real-time PCR instrument using the TB Green Premix Ex Taq II Kit (Takara Bio Inc., BeiJing, China). The reaction system (including cDNA template, specific primers, and master mix) was constructed according to the instructions of the kit. The relative expression level of the target gene was calculated according to the 2^−ΔΔCt^ method [56]; 3 biological replicates (independent samples) and 3 technical replicates were set up to ensure reliability. Primers used for qRT-PCR experiments are listed in Table 1.

### 4.6. Data Analysis

Microsoft Excel 2021 software was used to organise the data (Microsoft Corporation, Redmond, WA, USA). Origin 9.0 software was used to plot the data charts (OriginLab Corporation, Northampton, MA, USA). SPSS 27.0 was used to calculate and analyse the data (IBM Corporation, Armonk, NY, USA). LSD and Duncan methods were used to analyse the variance (ANOVA) to detect the differences between the groups. The Pearson correlation coefficient was used to identify correlations between data, and *p* < 0.05 was considered statistically significant.

## 5. Conclusions

Potato photosynthesis is the basis of potato tuber nutrition and yield formation; we studied the fluorescence parameters, photosynthetic gas exchange, and chlorophyll- and photosynthesis-related genes of potato plant leaf-related photosynthesis indexes, which are important indicators to measure the degree of alkaline salt stress. The fluorescence response *Fo* increased with the increase in stress concentration, while *Fv*, *Fm*, *Fv*/*Fm*, and *Fv*/*Fo* decreased significantly. The content of chlorophyll **a** and chlorophyll **b** gradually decreased as the stress concentration increased, with the chlorophyll **a**/**b** ratio significantly reduced. Notably, chlorophyll **b** exhibited a marked decrease of 54.3% compared to the control treatment. Under alkaline salt stress, the intercellular carbon dioxide concentration Ci in potato plant leaves initially exhibited a transient increase before gradually declining. Concurrently, photosynthetic parameters, including Pn, stomatal conductance Gs, and Tr were significantly suppressed. Compared to the control group, Pn, Gs, and Tr decreased by 63.6%, 83.4%, and 54.6%, respectively. A series of photosynthetic data measurements showed that the expression levels of ferredoxin Fd and FNR-related genes in the photosynthetic transport chain were significantly down-regulated by the high concentration of NaHCO_3_^−^ related ions. Moreover, alkaline salt stress markedly suppressed the expression of photosynthesis-related genes, thereby severely impairing the photosynthetic performance of potato plants. Results indicated that all sixteen photosynthesis-related genes exhibited varying degrees of down-regulation. Concurrently, this study elucidates how alkaline salt stress significantly inhibits photosynthesis by disrupting photosystem function and interfering with core physiological processes such as carbon fixation, through constructing a photosynthetic pathway map of potatoes under NaHCO_3_ stress. Further qRT-PCR analysis of expression levels in 16 key photosynthesis-related genes confirmed the severe damage inflicted by alkaline stress on photosynthetic mechanisms. This study is also helpful to understand the photosynthetic response mechanism of potato under alkaline salt stress and can also provide rich data support for subsequent research on potato variety improvement.

## Figures and Tables

**Table 1:** Gene IDs and primer sequences for the genes used for qPCR verification.

Gene ID	Forward Primer	Reverse Primer
PGSC0003DMG400010035	AGCCAACATCATTCACCGTCAAGG	GAACACGCTCACCACCAGGAAG
PGSC0003DMG400021727	TCTGGTGGATTGCCTGGAACTTTG	TCTGTGCTTCATCGCTGTTCTTGG
PGSC0003DMG400022241	GAAAGGGCTTCCATCACTTGCTAGG	CATCACCACTTGGAGACCACTCATC
PGSC0003DMG400017556	TGGGAGAAGCAATAACAGGAAAGGG	GAAGGATTTGCCAGGAGGGATGAC
PGSC0003DMG400011816	TCTCAGTAACCCGACCCATCCG	CAGCACCACCTGTTGGCATCTC
PGSC0003DMG400021144	TGTTGTCAGCACCAGTTCTTCCAG	TCCTTCCTACCCATCCAATCCATCC
PGSC0003DMG400016504	ATGGCGTCTATGGCAACTCTTACTG	TCTGAAGGGAGTTGTAGGGTGATGG
PGSC0003DMG400027672	TACCTCTCCAACTTGCCTGCCTAC	CTGGTGCTGTTGATGGATCTCCTTC
PGSC0003DMG400005805	TCACCACTGCTTCTTCTGCTAATGC	TCGCACTCCAACGCCAAATCG
PGSC0003DMG400005890	AAGACTAGCCTCTCCTCAGACTTCG	AGCACACCATCCATAGCCAGAATTG
PGSC0003DMG400023985	AAGTCTTCGCCCTCGTTCAAAGTC	CCATCTGATCATCGTCCAGGAATGC
PGSC0003DMG400026360	ATGTTCAAAGCCGCACCTCTGAG	AGCACAAGTTGAGCAAGCACCAG
PGSC0003DMG402005881	GCATCAGCATCAGCAGTTTACAAGG	GAGCACATAGCCCTCCTCCATTTG
PGSC0003DMG400011811	CTCTCAGGTGTCAGTTGCTGTTCC	TTGTCGCTGTGTATGGTTCCTTGG
PGSC0003DMG400016959	CGACGATGCGGTTTACGATTTCTTC	AGTCACCACAGCAAGTTCAGTATCAG
PGSC0003DMG400025106	TGAGAATACGCCTTAACGACCAATGG	AGCCTCCACCCGTGTTCTAGC

**Note:** Primer sequences for quantitative real-time PCR (qPCR). The second column lists the Forward primers and the third column lists the Reverse primers.

## Data Availability

The datasets generated and analysed during the current study are available in the NCBI repository, https://www.ncbi.nlm.nih.gov/sra/?term=SRP298983 (11 March 2025), the accession number is SRP298983. The datasets used and analysed in the current study are available from the corresponding author on reasonable request.

## Abbreviations

The following abbreviations are used in this manuscript:

PSII	Photosystem II
PSI	Photosystem I
ATP	Adenosine Triphosphate
NADPH	Nicotinamide Adenine Dinucleotide Phosphate, Reduced
NPQ	Non-Photochemical Quenching
OEC	Oxygen-Evolving Complex
PC	Plastocyanin
PQH_2_	Plastoquinol
ROS	Reactive Oxygen Species
QA	Quinone A
b_6_f	Cytochrome b_6_f Complex
NADP^+^	Nicotinamide Adenine Dinucleotide Phosphate, Oxidised
Rubisco	Ribulose-1,5-bisphosphate Carboxylase/Oxygenase
